# Characteristic of Apparent Diffusion Coefficient and Time Intensity Curve Analysis of Dynamic Contrast Enhanced MRI in Osteosarcoma Histopathologic Subtypes

**DOI:** 10.7150/ijms.77906

**Published:** 2023-01-01

**Authors:** Rosy Setiawati, Bagus Novariyanto, Paulus Rahardjo, Sjahjenny Mustokoweni, Giuseppe Guglielmi

**Affiliations:** 1 Radiology Department, Faculty of Medicine, Universitas Airlangga, Surabaya - Dr Soetomo General Academic Hospital Surabaya, Indonesia; 2 Department of Anatomical Pathology, Faculty of Medicine, Universitas Airlangga, Surabaya - Dr Soetomo General Academic Hospital Surabaya, Indonesia, Indonesia; 3 Department of Radiology, School of Medicine, Foggia University, Foggia, Italy

**Keywords:** Osteosarcoma, Apparent Diffusion Coefficient (ADC), Slope Time Intensity Curve (TIC), Osteosarcoma histopathology

## Abstract

**Background:** According to WHO criteria, osteosarcoma (OS) consists of various histopathological subtypes. Thus, contrast-enhanced MRI is a very useful modality in the diagnosis and evaluation of osteosarcoma. Magnetic resonance imaging with dynamic contrast enhancement (DCE-MRI) studies was used to determine the apparent diffusion coefficient (ADC) value and the slope of the time-intensity curve (TIC). This study aimed to determine the correlation between ADC and TIC analysis using %Slope and maximum enhancement (ME) of histopathological osteosarcoma subtypes.

**Methods:** This was a retrospective study with observational analysis on OS patients. The obtained data were 43 samples. Moreover, the interpretation was conducted by placing three regions of interest (ROI) in determining ADC value. It was observed by two radiologist observers with more than 10 years of experience. In this case, as many as six obtained ROIs were averaged. The inter-observer agreement was evaluated by Kappa test. TIC curve was analyzed and slope value was obtained afterward. Through SPSS 21 software, the data was analyzed.

**Results:** The mean of ADC values of OS was (1.031x10^-3^±0.31mm^2^/s), where the highest value was found in chondroblastic subtype (1.470 x10^-3^±0.31mm^2^/s). However, the mean of TIC %slope of OS was (45.3%/s), where the highest result was found in the osteoblastic subtype (70.8%/s) followed by small cell subtype (60.8%/s) and the mean of ME of OS was 100.55% with the highest values was in osteoblastic subtype 172.72% followed by chondroblastic subtype (144.92%). This study found a significant correlation between the mean of ADC value and the OS histopathologic results as well as the correlation between the mean of ADC value and ME.

**Conclusion:** The various types of osteosarcoma have a characteristic of radiological appearances which may similar to some bone tumor entities. The analysis of ADC values and TIC curves using % slope and ME of osteosarcoma subtypes can improve the accuracy of diagnosis as well as the monitoring of the treatment response and the disease progression.

## Introduction

According to WHO criteria, osteosarcoma (OS) consists of various histopathological subtypes 1. Magnetic resonance imaging (MRI) is an important imaging modality for preoperative and post-treatment evaluations for osteosarcoma. On MR imaging, conventional OS is defined as an aggressive bone intramedullary lesion with the characteristic of osteoid formation. There are some other non-conventional OS subtypes which are less common and may mimic other bone tumors on imaging. Moreover, conventional MRI is primarily indicated for local staging of the tumor and may not provide a specific histological diagnosis, especially in determining the extent of tumor necrosis or the presence of viable cells.

A potential impact of tumor heterogeneity is also the key of quantitative analysis of bone tumor. As a consequence, the requirements for spatial resolution arise because tumor vascularity is heterogeneous even at the microscopic level. It is important to considerate the contrast kinetics in a single voxel. Moreover, the contrast kinetic curve for a given voxel is the sum of all the curves originally from the smaller homogeneous, with well-mixed volume elements comprised 2. In the previous study, Tofts et al introduced the T1-weighted DCE Imaging concepts using repeated T1-weighted images which are collected for several frames before gadolinium is injected, then, for several minutes afterwards. This is preceded by a T1 measurement of all properties of each tissue voxel (e.g. T1, T2, diffusion tensor, magnetization transfer, metabolite concentration, K^trans^) 3,4. Therefore, the advanced MRI techniques for bone tumors are developed.

The advanced MRI techniques usually include; Dynamic Contrast Enhancement (DCE) perfusion imaging, Diffusion Weighted Imaging (DWI), Apparent Diffusion Coefficient (ADC) values, and MR spectroscopy 5. Prognostic and treatment response of osteosarcoma patient's evaluation are also the important aspects. Thus, the combination of advanced MRI techniques and conventional MRI is needed to increase the accuracy of diagnostic 5,6,7,8,9. Advanced MR imaging techniques enable the assessment of treatment-related response with the use of the presurgical study. It is a potential offer for standard treatment response criteria in which it will traditionally measure the changes in tumor size between the pre-treatment and post treatment images using Response Evaluation Criteria in Solid Tumors/RECIST) 10. It relates to the fact that tumor could die but not shrink. Moreover, the comprehensive analysis of anatomical and functional MR imaging sequences, compared with histopathologic examination, is a non-invasive and easily integrated with a routine presurgical examination. DCE MR imaging has also been studied for the assessment of treatment response in the patients with musculoskeletal tumors, but mainly in osteosarcomas 5,7,8,9,11.

ADC values from advanced MRI study could differentiate osteosarcoma with its differential diagnosis from other bone tumors e.g. Ewing's sarcoma 7,9,12. On the other hand, in the study by Yakushiji et al., it was difficult to distinguish osteosarcomas from chondrosarcomas, those tumors showed a restricted diffusion in DWI, while ADC value was considerably low. Thus, the use of water diffusion was embedded as a surrogate marker to characterize a highly cellular regions of tumor versus acellular and necrotic regions 6,7,8,9,12. The increased diagnostic accuracy may possible if DWI is used in conjunction with morphological gadolinium enhanced on MRI 12.

Furthermore, dynamic contrast-enhanced (DCE) of MR perfusion study is an evolving technology which able to give additional diagnostic information. It also provides a non-invasive assessment of microcirculatory characteristics of a lesion. Capillary blood flow, volume (relative or absolute), the uptake as well as the clearance of contrast medium (CM) from tissue are the functional parameters of tissue microcirculation 3. In addition, the total gadolinium concentration in a voxel or ROI is the sum of the extra-vascular extra-cellular space (EES) contribution. It usually becomes dominate because V_e_ approximately equals to 10-60% and the contribution of intravascular. The quantitative parameters e.g. K^trans^ and V_e_ may often be more sensitive markers of tumor metabolism 4,10.

In a basis, the curves were obtained by plotting the SI. Moreover, it provides qualitative and semi quantitative information and evaluates the lesions by determining Time Intensity Curve (TIC) slope value which changes over time. It is calculated by observing the percentage of the increase of signal intensity at initial tumor contrast enhancement (% per minute). It reflects the passage of the contrast agent within the target tissue 13. This technique has several important advantages i.e.; the ability to assess tissue characterization, local staging determination, active tumor areas identification for guiding biopsy, preoperative chemotherapy monitoring, residual or recurrent tumors detection, and differentiating tumors from fibrosis 14.

The presentation, prognosis, and therapeutic options may differ among OS subtypes. This study aimed to describe the advanced MRI feature of different OS subtypes and to analyze the correlation between ADC value and TIC analysis using %slope and ME.

## Materials and Methods

### Study Design

This prospective study was conducted in accordance with the Declaration of Helsinski. This study was approved by Medical Research Ethics Committee of Dr. Soetomo General Academic Hospital, Surabaya, East Java, Indonesia. All participants included in the samples have given their written informed consent to participate in this study during admission.

### Data Collection

In this study, from July 2019 to July 2021, 43 consecutive patients were observed (29 males and 14 females with an age range between 10 to 55 years and average age ± 22.511 years). Those suitable patients were observed within DWI and DCE MRI study. The inclusions were those who met the criteria of osteosarcoma patients who completed bone tumor MRI protocols including DWI-ADC and DCE MRI sequences prior to surgery. In addition, they were confirmed by histopathologic result (33 surgical biopsies and 10 percutaneous core biopsies). On the other hand, the exclusions were suited to the following criteria; the MRI data which were provided after neoadjuvant chemotherapy or radiotherapy and the suboptimal image quality of the MRI regarding to the patient's uncooperativeness and artefacts implantation.

### Imaging Protocol

The study protocols were performed by using Siemens Magnetom Skyra 3 Tesla MRI machine with; coronal, sagittal and axial sections, T1WI, T2WI, STIR sequences, DCE-MRI, DWI sequences, and ADC. On the other hand, DWI and ADC maps were observed on axial plane with b value 800 s/mm^2^. Formerly, contrast injection was performed using SS-EPI technique with the following parameters: TR (4430-6640 ms), TE (55-76 ms), FOV 200-325 mm^2^, matrix size (voxel) 115x128, 5-6 mm thickness with 1.5 mm interslice gap, and average of 1-2. Furthermore, DCE MR imaging was performed in the coronal or sagittal plane (time-resolved angiography with interleaved stochastic trajectories: 2.2-4.16/0.77-1.33; field of view, 230-400 mm; matrix, 108-256 pixels; section thickness, 3-8 mm, (based on the anatomic body part), capturing arterial, mixed, and venous phase images. The sequence was started simultaneously with an intravenous bolus administration of 0.1 mmol/kg bwt. Gadolinium-DTPA injected at a rate of 3 mL/sec and images from 30 phases were acquired within a minimum temporal resolution of time as in seven second. Moreover, a composite set of images was reconstructed with maximum intensity projection in coronal, axial, and sagittal planes. The enhancement in the volume of interest could be viewed throughout different phases.

### Imaging Interpretation

**Figure 1:**
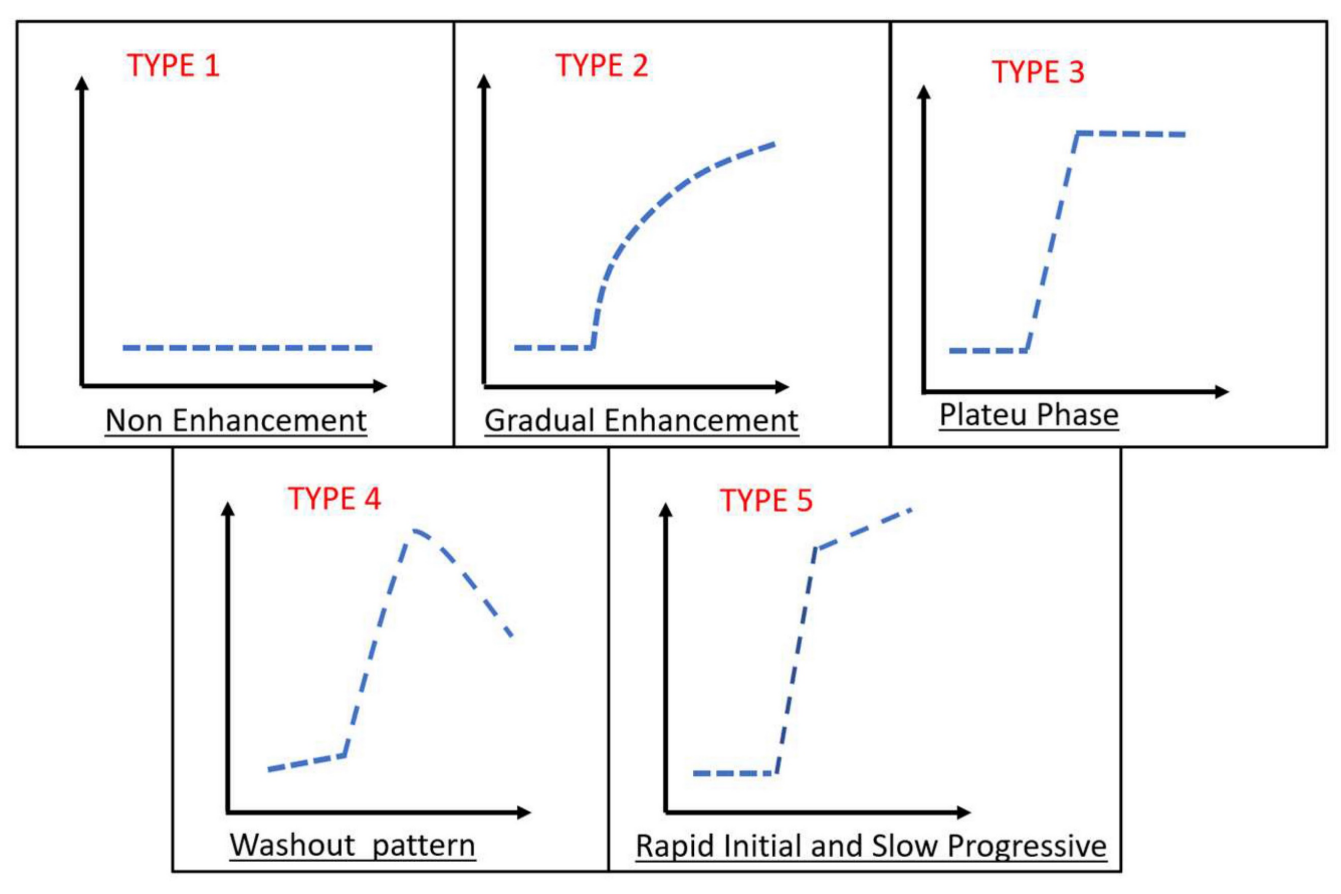
Types of Time Intensity Curves (TICs). Type I: no enhancement; type II: gradual increase of enhancement; type III: rapid initial enhancement followed by plateau phase; type IV: rapid initial enhancement followed by washout phase; type V: rapid initial enhancement followed by slow progression of enhancement

The interpretation of ADC value was performed by two observers i.e. musculoskeletal radiologist consultants with more than 10 years of experience. They reviewed the arterial phase images of the DCE MR imaging sequence (defined as the image on which arterial filling was first identified) and qualitatively recorded the presence or absence of early tumor enhancement. The ROIs placement of each tumor images was performed on adequate tissues. Therefore, it contained the most restricted on DWI only, enhanced solid components on DCE, and post contrast images avoid areas that may influence the ADCs. Necrotic, fibrotic and haemorrhagic areas as well as adjacent fat, normal tissue and bone were also influenced. The ROI shape was taken as a round or an oval with a minimum area of ​​five mm^2^ and a maximum area of 25 mm^2^. In order to collect the ADC values and determined the mean of ADC values, three ROIs intra lesion were obtained. The inter-observer agreement was evaluated by Kappa test. From DCE-MRI study, the TIC type was determined qualitatively and TIC % slope and maximum enhancement value was calculated afterward. The schematic of the different time intensity curve types was described in **Figure 1**. The slope of the curve (the percentage increase in signal intensity per minute over the baseline value) was derived by using the following equation:

%Slope (%/s) = (SI max - SI base) / (SI base x (Tmax - Tbase)) x 100

SI max: signal intensity on Tmax

SI base: signal intensity on Tmin

Tmax: time on maximum intensity

Tmin: time on minimum intensity/start enhancement

**Figure 2:**
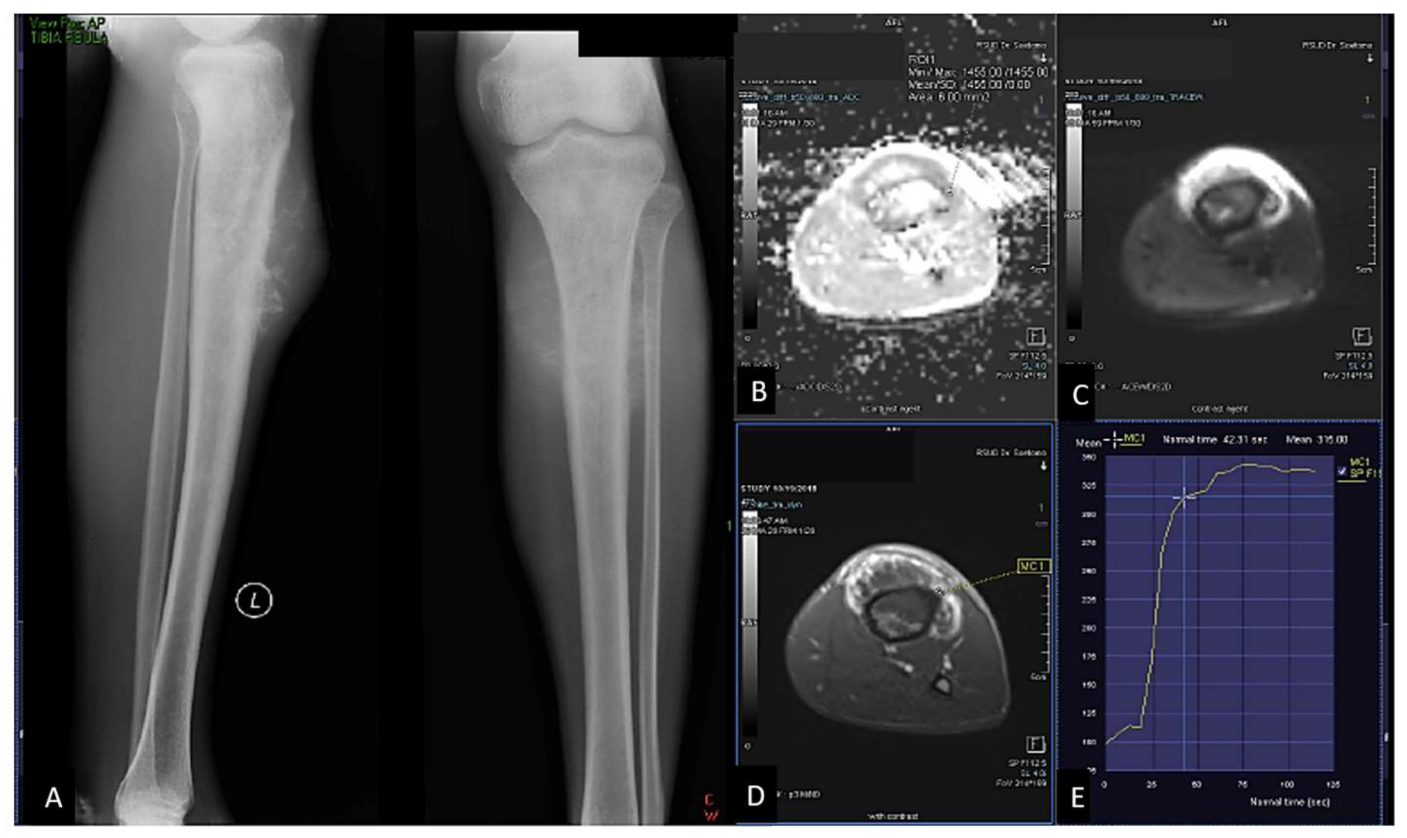
23 years-old man, with chondroblastic osteosarcoma histopathologically in proximal left tibia. X-ray left cruris AP and lateral projection (A) multiple osteolytic lesion in diaphysis of proximal left tibia with chondroid matrix and soft tissue bulging around it. Restricted diffusion on DWI and ADC map (B,C) with ADC value 1.245 x 10-3 mm2/s, on Dynamic Contrast Enhancement show early contrast enhancement followed by plateu phase /TIC type 3 (D,E) with the %slope result was 7,52 %/s.

**Figure 3:**
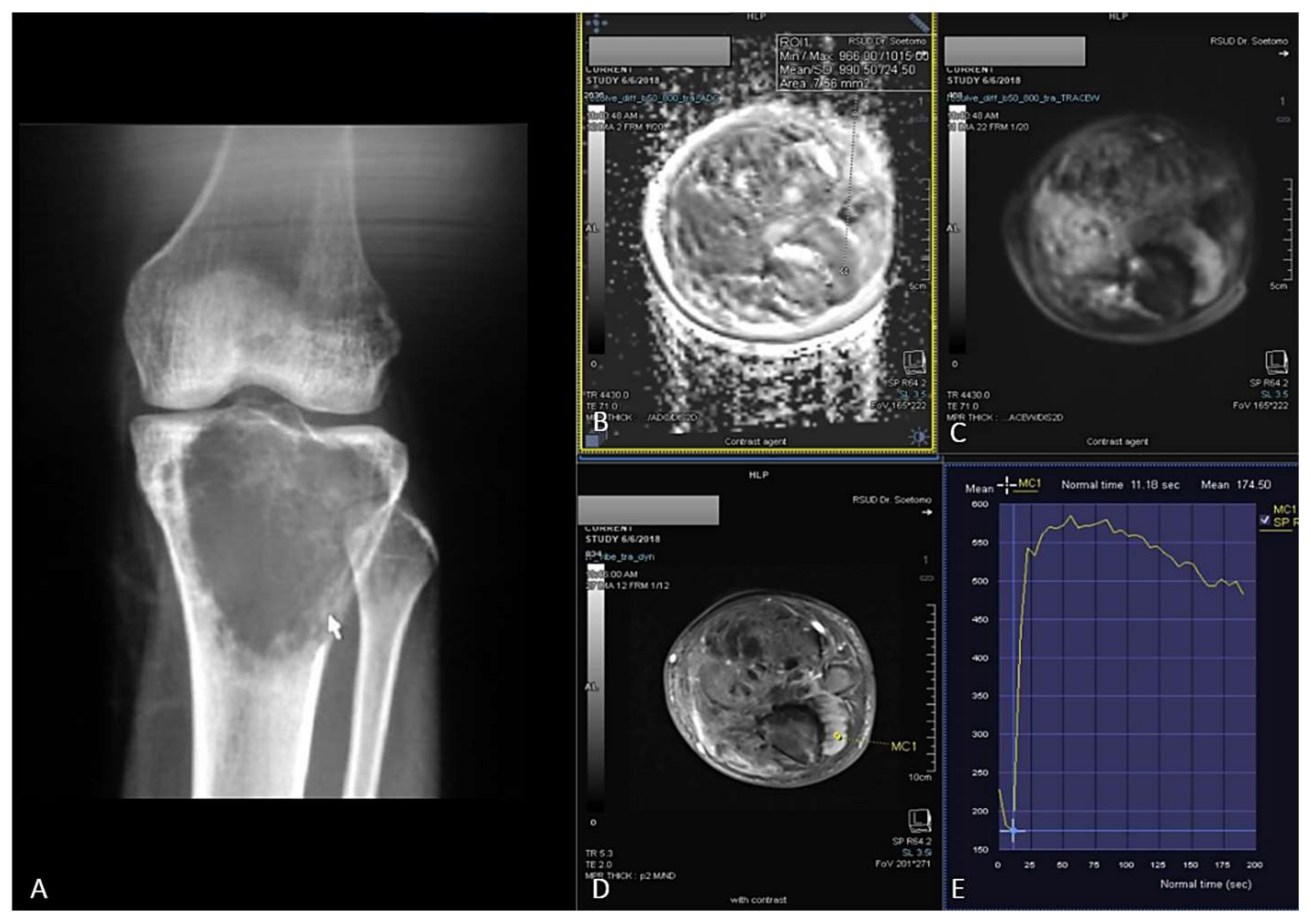
19 years-old man, with osteoblastic osteosarcoma histopathologically in proximal left tibia. X-ray left knee AP projection (A). A single ill-defined osteolytic lesion in diaphysis of proximal left tibia. Restricted diffusion on DWI and ADC map (B,C) with ADC value 0,955 x 10-3 mm2/s, on Dynamic Contrast Enhancement show early contrast enhancement followed by wash out pattern / TIC type 4 (D,E) with %slope result was 19,18 %/s.

In this study, TIC %slope measurement was based on the formula 5,11. Maximum enhancement is defined as maximum signal difference (MSD)/signal baseline (SB), where MSD is the difference between the signal intensity at its maximum (SI_max_) and SB. The sample of ROI placement of ADC value and DCE was shown in **Figure 2** and** Figure 3**.

### Data Analysis

Data analysis was performed using SPSS 23 statistics software. The mean of ADC value from each observer was tested for inter-observer agreement. The inter-observer agreement was evaluated by Kappa test, where p <0.05. Apparent Diffusion Coefficient and TIC values from MRI study and histopathological examination results were presented in the tables. The analysis for correlation between ADC values and TIC slope with histopathological subtypes was tested using Kruskal-Wallis test. Spearman's rho test was also used to determine the correlation between the mean ADC and ME value and between the TIC % slope and ME.

## Results

This was a retrospective study with observational analysis on 43 samples of the osteosarcoma patients. **Table 1** presented the characteristics of study sample. It could be inferred that the samples were predominantly in 29 male patients (67.4%), mostly in 11-20 years group with 23 patients (53.5%), and the common bone tumor locations were on distal femur as many as 21 (48.8%) and proximal tibia at 18 (41.8%).

From **Table 2**, the osteosarcoma histopathology results were categorized to various subtypes. In this study, we found that the most osteosarcoma subtypes were osteoblastic osteosarcoma (46.5%), followed by chondroblastic subtype (18.6%), and giant cell rich subtype (14.0%). The least type was low grade osteosarcoma with one patient found. Initially, the results in ADC value have been tested by Kappa test. They have been observed by two observers. It was found that the kappa value (κ) was 0.849 with p <0.05. The kappa result showed that the two observers had the same agreement in the extent of strong ADC value result. The mean of ADC value of osteosarcoma in this study was 1.035±0.31x10^-3^ mm^2^/s. The highest mean of ADC value was found in chondroblastic subtype (1.470±0.32 x10^-3^ mm^2^/s), followed by fibroblastic (1.003±0.25 x10^-3^ mm^2^/s) and osteoblastic subtype (0.994±0.24 x10^-3^ mm^2^/s). From the comparison between ADC values and the histopathologic subtypes, it showed that p=0.0023 and indicated that there was a significant difference between the mean of ADC value and the osteosarcoma subtypes.

This study presented the characteristics of TIC DCE-MRI in various osteosarcoma histopathological subtypes (**Table 3**). There were TIC type 3 in 26 patients (60.5%), while TIC type 4 in 17 patients (39.5%). The most TIC type 3 were found in chondroblastic subtypes (7 patients, 87.5%) and osteoblastic subtypes (12 patients, 60.0%), whereas; TIC type 4 were mostly found in telangiectatic subtypes (2 patients, 66.7%). All small cell subtypes samples had TIC type 4, while low grade osteosarcoma subtypes (only one sample) found in TIC type 3. The result of mean slope of osteosarcoma was also outlined in this study. The mean value of osteosarcoma in TIC % slope from DCE MRI was 45.3 %/s. The highest mean value of TIC % slope was found in osteoblastic OS (70.8 %/s), followed by chondroblastic OS (56.7 %/s) and fibroblastic subtype (43.0 %/s). On the other hand, the highest of maximum enhancement mean value was found in osteoblastic OS (172.72 %), followed by chondroblastic OS (144.95 %) with the lowest value found in a small cell OS (54.80 %).

In addition, the Shapiro-Wilk test was performed to assess the normality of the data distribution in identifying the mean of ADC value and TIC % slope for various osteosarcoma subtypes. Based on the test results, it was found that the data distribution was abnormal for the mean of ADC and TIC % slope, because the p value was <0.05. The correlation test between the mean of ADC value and the osteosarcoma histopathological subtype showed that p=0.0001. It indicated that there was a significant correlation between the mean of ADC value and the osteosarcoma subtypes. Meanwhile, the correlation test between the mean TIC slope and the histopathological subtype of osteosarcoma showed that p=0.323.

It could be inferred that there was no significant correlation between the mean of TIC% slope and the osteosarcoma histopathologic subtypes. The correlation between the mean of ADC value and ME value showed that p = 0.000 with a correlation coefficient (r) = 0.864. It indicated that there was a significant correlation between the mean ADC value and ME. Otherwise, the correlation between the mean of TIC % slope and ME showed that p = 0.133. This result confirmed that there was no relationship between the mean value of TIC % slope and ME.

## Discussion

The emergence of magnetic resonance imaging (MRI) plays the important role in the extent of diagnostic method for local staging of primary bone tumors and for postoperative tumor recurrence detection. MRI allows the accurate preoperative staging of local tumor extension and helps to obtain the adequate safe tumor margins. Furthermore, it is a prerequisite notion for a limb salvage operation to be successful. Conventional MRI will provide clinically important information about tumor volume and area; however, it provides insufficient information about tumor survival rates, important parameter in prognosis, and determining tumor response. In some cases, it may not provide a specific histological diagnosis, especially in determining the tumor necrosis or highlighting the presence of viable cells 5,6,14. The combination of advanced MRI techniques and conventional MRI can increase the accuracy of diagnostic, prognostic and treatment response evaluation 15. This study aimed to determine the correlation between Apparent Diffusion Coefficient (ADC) value and slope Time Intensity Curve (TIC) with osteosarcoma histopathologic subtypes.

It could be inferred that the age characteristic which was exposed mostly in second decade of life, 11-20 years group to be exact. At this age, the fastest bone growth occurred. Osteosarcoma has a predilection for developing in rapidly growing bone. A number of studies have identified that there was a correlation between the experienced rapid bone growths and the human puberty. Moreover, people in second decade of human's life are experienced a puberty phase. In this case, bone develops rapidly in regard to osteosarcoma development 15,16,17. The location of our samples was found commonly in distal femur and proximal tibia. Fifty-six percent of all osteosarcomas present around the knee joint, consistent with the epiphyseal growth plates of the distal femur and proximal tibia. They have a higher responsibility for a great deal of the increase in the height that occurs during puberty 18,19,20.

The lowest mean of ADC value in this study was found in osteoblastic subtype 1.035±0.31x10^-3^ mm^2^/s. This result was in concordance with a study from Zeitoun et al., they stated that higher cellular matrix osteoid caused low ADC value and was found in the osteoblastic OS 18. Another study conducted by Setiawati et al. in 2021 stated that the increase of osteoid matrix between chondroid matrices would increase DWI restriction and decrease ADC value 21. The highest mean of ADC value was found in chondroblastic subtype. This result was similar to a study from Yakushiji et al., in the extent of the tumor cells are more freely dispersed among the chondroid matrices in chondroblastic osteosarcoma 22,23. This study pointed to lower ADC values for osteoblastic, small cell, giant cell rich type OS lesions reflecting higher cellularity of the tumor osteoid matrix, small round cells and giant-cell stroma respectively.

Osteosarcoma is a primary malignant bone tumor that produces matrices e.g. osteoid, chondroid or fibrous, and the largest composition is the osteoid matrix. The differentiation in osteosarcoma will determine the histopatological subtype 21. In this study, the most predominant osteosarcoma was osteoblastic subtype. Osteoblastic subtype had the lowest mean of ADC value, where it was 0.994x10^-3^ mm^2^. Meanwhile, chondroblastic subtype had the highest mean of ADC value, 1.47x10^-3^ mm^2^. This result corresponded with previous study from Zeitoun et al., they stated that the highest ADC value was osteoblastic subtype and high cellular matrix osteoid resulted in restricted diffusion and low in ADC value 18. Another study conducted by Setiawati et al. in 2021 stated that the increase of osteoid matrix between chondroid matrices would increase DWI restriction and decrease ADC value 21. In this study, the highest mean of ADC value was found in chondroblastic subtype. This result was similar to study by Setiawati et al. in 2021, where the mean of ADC value in malignant chondrogenic bone tumor was 1.84x10^-3^ mm^2^. Moreover, another study by Yakushiji et al. in 2009 stated that chondroblastic osteosarcoma had a higher mean of ADC value than other osteosarcoma subtypes 21,23. Tumor cells are more freely dispersed among the chondroid matrices in chondroblastic osteosarcoma, cause a decrease of the restricted diffusion, and an increase of ADC value 18,21,24 .

Semiquantitative analysis could be applied by using a first-pass method. The first-pass method assumed that the dynamic enhancement pattern that was observed during initial first-pass (slope) will mainly represent the contrast agent kinetics in blood vessels; however, the peak of TIC will represent the enhancement induced by contrast agents in both the intravascular and extravascular spaces 21,25. In this study, the mean of TIC %slope and ME values of osteosarcoma on DCE-MRI study were 45.3%/s and 100.55% with the highest values of both TIC %slope and ME were found in osteoblastic subtype (70.8%/s and 172.72%). Osteoblastic OS subtype has much vascularization compared to other subtypes. The highest quantitative of TIC results using %slope and ME representation covered its steepest slope value which accurately reflected tumor vascularization. These results were similar to previous studies conducted by Varidha and Setiawati in 2020, they reported that the mean slope of osteosarcoma was 68.59%/s 26. Yong Park et al (2014) stated that the steepest slope and early relative elevation signal intensity have a potential role in differentiating benign and malignant soft tissue tumor 27.

In general, malignant tumor has much vascularity and narrow extravascular or interstitial space. Thus, it exhibited rapid and high contrast enhancement 2,25. Tucbilek et al (2004) stated that the steepest lope value accurately reflects tumor vascularization, the malignant soft tissue tumor showed early elevation signal intensity and steepest slope e.g. liposarcoma 28. Two cases of chondroblastic and osteoblastic OS with the presented of ADC value mapping and TIC on DCE were described on **Figure 1** and **Figure 2**.

In this study, the sample characteristics were predominantly male in 11-20 years group, with tumor locations predominantly found in distal femur and proximal tibia. The characteristics of our samples corresponded with the typical osteosarcoma characteristics. The lower mean of ADC value in type 3 and 4 TIC patterns showed the malignancy characteristic of a lesion. In addition, the correlation between the mean of ADC value and osteosarcoma histopathological subtypes may increase the imaging diagnostic accuracy of osteosarcoma. The TIC %slope value may also assist the analysis to establish osteosarcoma diagnosis. The strong relationship between the mean value of ADC and ME found in this study was in accordance with the principle of contrast enhancement in producing a TIC curve. It was the total volume of accumulated contrast agent in tumor vascular and interstitial space (EES), reflected in the maximum increase between changes in tumor signal intensity before and after enhancement 27,28,29. The osteosarcoma subtypes exhibited the distinct radiologic features that may be mimicked by various benign and malignant bone tumor entities. Therefore, MR perfusion and diffusion was a beneficial way in distinguishing the characteristics of each OS subtype 30.

Regarding to the pattern of ADC value and semiquantitative TIC curve analysis, it is a helpful aspect to determine the variety tissue and matrix characterization of osteosarcoma histopathologic subtypes. The disease progressiveness and prognosis are also the key point. It may contribute in predicting the treatment response 29,31. Conventional osteoblastic OS subtypes have a tendency to behave more aggressively as in the extent of intra-articular extension, invasion neighbouring bone, and the incidence of pathological fractures and distant metastases. All OS subtypes exhibit an ossification matrix and represent as a variable proportion in the area of ​​the hypointense signal in all pulse-weighted images. They become the most prominent in the conventional osteoblastic OS 18,32. According to the Salzer-Kuntschik classification, the treatment response is proper when viable tumor cells are histologically less than 10%, based on the type of 1 to 3 31.

The study conducted by Bacci et al. pointed to the presence of significant correlation between OS histopathologic subtypes and their response to neoadjuvant chemotherapy. The rate of good therapeutic response (90% or more tumor necrosis) and 5-year survival were significantly higher in fibroblastic and telangiectatic OS, hence; the authors concluded that there was a correlation between OS subtype and the prognosis 32. Both dynamic contrast-enhanced MRI and diffusion-weighted MRI allow monitoring of perfusion changes induced by vascular targeting agents in tumors. Diffusion-weighted imaging provides additional information about intratumoural cell viability versus necrosis after chemotherapy administration. Due to water mobility within a tumor, it will increase over time after the treatment and the magnitude of change would be related to the effectiveness of therapy. As a result, there would be a membrane damage and subsequent reduction in the cell density 6,14,33.

This study has some limitations. Firstly, the sample distribution is not comprehended for each osteosarcoma subtype group due to its rarity. Moreover, most of the samples showed a large tumor size with heterogeneous component. Thus, the wide variety of the ADC values and slope of TIC might be occurred. Secondly, the area of ROI placement which will be used to obtain ADC value and TIC analysis could not be adapted to the same histopathological specimen sampling area.

## Conclusion

There was significant correlation between the mean of ADC values and osteosarcoma histopathological subtypes as well as ADC values and ME. In addition, the TIC slope may also assist in establishing the analysis of osteosarcoma diagnosis. The osteosarcoma subtype exhibits distinct radiologic features, prognosis, treatment response and patient survival. Thus, the knowledge of ADC value and semiquantitative TIC curve analysis increases the accuracy of osteosarcoma diagnosis.

## Author contributions

Rosy Setiawati: Conceived and designed the analysis; Contributed data or analytic tools; Performed the analysis; Wrote the paper.

Bagus Novarianto: Collected the data; Contributed data or analytic tools; Wrote the paper.

Paulus Rahardjo: Collected the data; Contributed data or analytic tools; Performed the analysis.

Sjahjenny Mustokoweni: Conceived and designed the analysis; Performed the analysis; Wrote the paper.

Giuseppe Guglielmi: Conceived and designed the analysis; Contributed data or analytic tools, Performed the analysis; Wrote the paper.

## Figures and Tables

**Table 1:** Characteristics of Study Samples

No	Variables	Samples (*n*=43)
1	**Age Group**	
	0-10 years	3 (7.0%)
	11-20 years	23 (53.5%)
	21-30 years	9 (20.9%)
	31-40 years	4 (9.3%)
	41-50 years	1 (2.3%)
	>50 years	3 (7.0%)
2	**Gender**	
	Male	29 (67.4%)
	Female	14 (32.6%)
3	**Bone Tumor Location**	
	Distal Femur	21 (48.8%)
	Proximal Tibia	18 (41.9%)
	Fibula	1 (2.3%)
	Humerus	2 (4.7%)
	Pelvic	1 (2.3%)

**Table 2:** Histopatological Subtype Distribution and ADC Value Result

Osteosarcoma Subtypes	Samples (n=43)	Percentage (%)	Minimum ADC Current Study (x10^-3^mm^2^/s)	Maximum ADC Current Study (x10^-3^mm^2^/s)	MeanADC value Current Study (x10^-3^mm^2^/s)		ADC value Zeitoun et al. (2018)^5^ (x10^-3^mm^2^/s)
Osteoblastic OS	20	46.5	0.585	1.245	0.994		1.01
Chondroblastic OS	8	18.6	1.080	2.082	1.470		1.32
Fibroblastic OS	3	7.0	0.825	1.288	1.003		1.12
Telangiectasis OS	3	7.0	0.894	0.946	0.924		1.36
Small Cell OS	2	4.7	0.689	0.781	0.735		1.11
Giant Cell-Rich OS	6	14.0	0.715	0.931	0.817		-
Low Grade OS	1	2.3	0.746	0.746	0.746		-

**Table 3:** Time Intensity Curve and Mean TIC % slope

Subtype	TIC Type		* Slope* (%/s)	*Mean Maximum Enhancement* (%)
	Type 3	Type 4		
Osteoblastic Osteosarcoma	12 (60.0%)	8 (40.0%)	70.8	172.72
Chondroblastic Osteosarcoma	7 (87.5%)	1 (12.5%)	56.7	144.92
Fibroblastic Osteosarcoma	2 (66.7%)	1 (33.3%)	43.0	88.86
Telangiectatic Osteosarcoma	1 (33.3%)	2 (66.7%)	22.8	71.33
Small Cell Osteosarcoma	0 (0.0%)	2 (100.0%)	60.8	54.80
GiantCell-Rich Osteosarcoma	3 (50.0%)	3 (50.0%)	32.2	109.65
Low Grade Osteosarcoma	1 (100.0%)	0 (0.0%)	30.8	61.59
The Mean value	-	-	45.3	100,55

## Abbreviations

ADC: Apparent Diffusion Coefficient
DCE - MRI: Dynamic Contrast Enhancement Magnetic Resonance Imaging
DWI: Diffusion weighted imaging
EES: Extra-vascular extra-cellular space
FOV: Field of view
Ktrans: The volume transfer constant
MRI: Magnetic resonance imaging
MSD: Maximum signal difference
OS: Osteosarcoma
ROI: Region of interest
SI max: Signal intensity maximum
SI base: Signal intensity base / minimum
STIR: Short Tau Inversion Recovery
TE: Echo time
TIC: Time Intensity Curve
T1WI: T1 weighted image
T2WI: T2 weighted image
Tmax: Time on maximum intensity
Tmin: Time on minimum intensity/ start enhancement
TR: Repetition time
Ve: The fractional volume of EES
